# Association of Myostatin Gene Polymorphisms with Strength and Muscle Mass in Athletes: A Systematic Review and Meta-Analysis of the MSTN rs1805086 Mutation

**DOI:** 10.3390/genes13112055

**Published:** 2022-11-07

**Authors:** Marek Kruszewski, Maksim Olegovich Aksenov

**Affiliations:** 1 Department of Physical Education, Faculty of Individual Sports, Jozef Pilsudski University of Physical Education in Warsaw, 00-968 Warszawa, Poland; 2 Academic Department of Physical Education, Plekhanov Russian University of Economics, Moscow 117997, Russia; 3 Department of Physical Education Theory, Faculty of Physical Training, Sport and Tourism, Banzarov Buryat State University, Ulan-Ude 670000, Russia

**Keywords:** myostatin, *MSTN*, muscle, strength, hypertrophy, hyperplasia, training, meta-analysis

## Abstract

Polymorphism (rs1805086), *c.458A>G, p.Lys(K)153Arg(R)*, (*K153R*) of the myostatin gene (*MSTN*) has been associated with a skeletal muscle phenotype (hypertrophic response in muscles due to strength training). However, there are not enough reliable data to demonstrate whether *MSTN* rs1805086 K and R allelic variants are valid genetic factors that can affect the strength phenotype of athletes’ skeletal muscles. The aim is to conduct a systematic review and meta-analysis of the association of *MSTN* rs1805086 polymorphism with the strength phenotype of athletes. This study analyzed 71 research articles on *MSTN* and performed a meta-analysis of *MSTN K153R* rs1805086 polymorphism in strength-oriented athletes and a control (non-athletes) group. It was found that athletes in the strength-oriented athlete group had a higher frequency of the *R* minor variant than that in the control group (OR = 2.02, *P* = 0.05). Thus, the obtained results convincingly demonstrate that there is an association between the studied polymorphism and strength phenotype of athletes; therefore, further studies on this association are scientifically warranted.

## 1. Introduction

### 1.1. History of Discovery

In addition to sports training, environmental exposure, nutrition, and professional activity of a person, genetic factors also have a great influence on the strength indicators of an athlete’s skeletal muscles [1]. The study of genetic foundations, including gene polymorphisms and their connections with a body’s resistance to physical load as a whole, and their contribution to an athlete’s strength abilities and development should reasonably be considered as one of the most important and significant areas of modern sports science [2].

Myostatin (*MSTN*) protein was discovered in 1997 and was encoded by the *MSTN* gene, located on chromosome 2 2q32.2; it encodes 375 amino acids in three exons and occupies a site of approximately 8 kb [3]. This discovery was considered a significant success in the study of genetic factors for increasing muscle mass and developing strength abilities.

This gene was named *MSTN* because of its ability to inhibit muscle differentiation and growth [4], whereas the overexpression of *MSTN* is associated with muscle atrophy [5]. However, these studies have confirmed the central and critical role of *MSTN* in suppressing muscle growth [6,7].

Special attention is paid to *MSTN* because the very first publications on this factor concluded its absence to affect an increase in muscle mass due to hypertrophy and hyperplasia of the muscle fibers [8]. The increase in the detailed scientific studies of *MSTN* and the possibilities of using the published data for various biomedical and sports purposes, including gene doping [9], has increased interest in the subject.

The ability of *MSTN* to limit the growth of muscle mass immediately attracted the attention of researchers, as it can be used in sports and sports medicine.

*MSTN*, also known as the growth differentiation factor-8 (*GDF-8*), is a protein-based hormone that acts as a negative regulator of muscle growth. This was first mentioned by McPherron et al. [10]. The authors found that a mutation in *MSTN* leads to an increase in the size of muscle tissue. In the initial stages, these were primarily conducted on animals followed by those on humans. *MSTN* is particularly of interest in sports, wherein one can monitor its correlation with performance, especially in sports that require muscle strength and mass [10].

Mutations in *MSTN* lead to a significant increase in muscle mass [11]. It is an important gene that affects myogenesis as its role is to regulate the growth and differentiation of muscle cells [12]. In particular, the genetic predisposition to gain muscle mass is due to the low expression of *MSTN*, which is advantageous in the improvement of strength [13].

As *MSTN* is the most common type of skeletal muscle protein, it is of interest in studies related to sports science [14]. However, its expression is also noted in the heart and adipose tissues [15,16].

**Figure 1:**
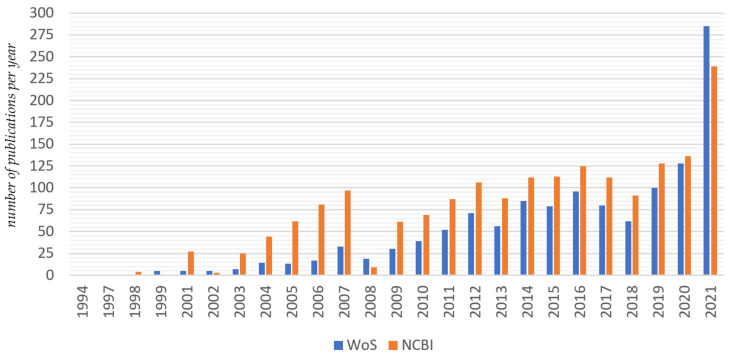
Dynamics of publications on *MSTN* (data as on December 2021).

The growing interest in *MSTN* has led to a large number of scientific publications that can be found in the Web of Science and PubMed database (Figure 1). A review of the publications confirms that *MSTN* is an endogenous negative regulator of skeletal muscle mass, which acts as an antianabolic agent that suppresses the activation, replication, DNA, and protein synthesis of muscle satellite cells affecting myogenic differentiation [17].

Researchers from Taiwan found that *MSTN* was negatively correlated with age and the percentage of fat mass in healthy young men [18]. The results of the experiments proved that the reference value of *MSTN* concentration in blood serum in healthy young men is 12.3 ± 3.6 ng/mL and that it negatively correlates with age [19].

Considerable attention should be paid to the factors contributing to the inhibition of *MSTN* expression. According to scientific sources, such factors include hypodynamia, various origin diseases, state of weightlessness, and aging [9,20]. The level of *MSTN* in skeletal muscles is also influenced by power-oriented physical exercise [21].

#### 1.1.1. MSTN Inhibitors

There are a number of factors that act as inhibitors of *MSTN* synthesis, including the myocyte 2 enhancing factor (*MEF2*); γ-receptors activated by peroxisome proliferator (*PPARγ*); MyoD; and hormones, such as insulin-like growth factor (*IGF-1*), angiotensin II, thyroid hormone, erythropoietin [22], sex steroids, follistatin, and estradiol [23].

One of the main factors in sports that significantly affects the level of *MSTN* secreted is power-oriented physical activity, hypoxia, and dietary supplements. Moreover, the production of *MSTN* is influenced by essential amino acids, which are often consumed by athletes after intensive training [24].

Currently, the study of antibodies against *MSTN*, e.g., MYO—029 and BYM338, are attracting much attention, but their effectiveness is still poorly studied [25,26]. In addition to antibodies, other *MSTN* inhibitors, such as hormone follistatin, can also suppress its activity [27,28,29].

Recent studies have shown that essential amino acids suppress *MSTN* expression in human skeletal muscles [30,31].

In high-performance sports, *MSTN* inhibition is prohibited by WADA (https://www.wada-ama.org/en/resources/world-anti-doping-program/2023-prohibited-list, accessed on 15 October 2022; page 11 class S4).

*MSTN* is a potential genetic marker of the athletic abilities in strength sports because of the involvement of a large number of skeletal muscles and the functions of myokines. Some research related to the study of *MSTN* and its role in hypertrophy and skeletal muscle strength seemed contradictory [20,32,33]. Therefore, in this study, attention was given to the influence of the *K153R* rs1805086 polymorphism on the manifestation of skeletal muscle strength in athletes.

#### 1.1.2. Mechanism of Effect of *MSTN* on Skeletal Muscle Mass and Strength

Physical activity causes muscle hypertrophy and performing physical power-oriented exercises clearly demonstrated this. This type of exercise causes mechanical damage to sarcomeres and sarcolemmas. After a certain period of time, the balance shifts toward protein synthesis and, as a result, phenotypic changes increase the volume and strength of skeletal muscles. These processes release active *MSTN*, which affects satellite cells and fibroblasts located near the damaged area. *MSTN* can cause protein degradation in myofibrils, which are important for the normal functioning of muscle fibers as they remove unnecessary, wasted proteins from the muscle cells [34].

*MSTN* is one of the main factors associated with muscle atrophy. In studies involving humans, it was found that by the 25th day of the sedentary regime, the level of *MSTN* increased by 12% [9]. *MSTN* can regulate the function of muscle fibers and nearby cells, which include fibroblasts and satellite cells or satellites. Mature muscle fibers are the products of final differentiation [28].

**Figure 2:**
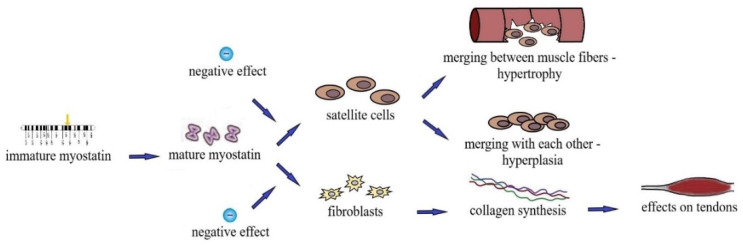
Molecular mechanisms of athletes’ strength abilities.

An increase in muscle size is achieved by the fusion of satellite-proliferating cells with fibers. Primarily, microtrauma in a single muscle fiber acts as a stimulus for the proliferation of satellite cells in adult organisms. When these cells are activated and emerge from a dormant state, genes characteristic of myoblasts are also activated. Therefore, satellite cells become myoblasts that migrate to the damaged areas of muscle tissue and depending on the degree of damage, either merge with the damaged muscle fiber (hypertrophy) or merge with each other, thus creating new fibers (hyperplasia). Therefore, satellite cells regulate the functional state of skeletal muscles in the adult body (Figure 2). They are necessary for the restoration of damaged muscle fibers and are a source of additional nuclei in case of muscle hypertrophy after training sessions. *MSTN* negatively affects the proliferation of satellite cells [35]. Power-oriented training sessions result in mechanical stretching of the muscle and lead to microdamage. There is also evidence that *MSTN* negatively regulates the activation of resting satellite cells, hindering their development. Such inhibitory effects are necessary for normal muscle regeneration as a premature fusion of satellite cells with myofibrils can impair muscle fiber functions.

In general, the mechanism by which *MSTN* controls the number of muscle fibers is not well studied. It is synthesized as an inactive protein and undergoes changes to turn into a mature active form in two stages [36]. It enters the bloodstream as a latent precursor protein and undergoes a proteolytic process, turning into a mature peptide that binds to the extracellular type II receptor (*ActRIIB*) activin. The binding of *MSTN* to *ActRIIB* induces the intracellular activation of proteins, by which *MSTN* modulates the proliferation and differentiation of myoblasts, and ultimately, the muscle mass [30,37,38].

#### 1.1.3. Effect of MSTN on Tendons and Bones

Tendons are an important component in the manifestation of the maximum strength of the skeletal muscle. Weightlifting and speed-strength sports athletes with high indicators of skeletal muscle strength often have tendon injuries as their muscle strength exceeds their endurance. During strength training, fibroblasts proliferate, collagen synthesis increases and the cross-sectional area of the tendons increases to make them stiffer. This allows the tendons to withstand high-intensity physical loads and reduce the risk of damage to them [39].

*MSTN* can change the mechanical properties of tendons by impairing their ability to stretch, increasing the risk of damage. Such data cast doubt on the feasibility of inhibiting *MSTN* expression for sports purposes [35]. The exact mechanisms of the effect of *MSTN* on tendons and ligaments are still unknown, and further studies are needed to assess its regulatory role in these processes [40]. When studying the regeneration of muscles and tendon fibroblasts, it is assumed that *MSTN* affects the expression of type 1 collagen. Recent studies have reported that local injections of exogenous *MSTN* during tendon healing increase the cross-sectional area of the tendon [41].

In both human and animal studies, there is evidence that *MSTN* is an important regulator of muscle mass as well as bone density. The mechanisms by which *MSTN* regulates bone formation are not completely understood, but it is clear that it has a direct effect on the proliferation and differentiation of stem cells [42,43]. Since *MSTN* and its receptor are expressed during bone regeneration, it affects bone density [43]. It is likely that *MSTN* directly affects bones, increasing bone mineral density. Some features of different phenotypes may be associated with increased biomechanical load, e.g., in weightlifters or under the influence of other factors, such as mechanical growth factors or growth hormones. These issues have yet to be studied in more detail, but if a number of studies prove that *MSTN* does have an effect on bones, then it can be assumed that *MSTN* inhibitors will be useful not only for increasing muscle mass but also for bone density. This assumption is supported by recent data showing that *MSTN* significantly increases bone volume during fibular healing [44].

### 1.2. Myostatin Mutations

In previous studies, it was found that a number of missense substitutions in exons 1 and 2 of *MSTN* are of great interest to researchers to confirm *MSTN* connection with athletes’ strength abilities, muscle hypertrophy [45], and recovery after intensive strength exercises. The polymorphisms *K153R*, *A55T*, *E164K, P198A*, *I225T*, and *c.373 + 5* [20,46,47,48] are of particular interest to the gene as well.

#### 1.2.1. *MSTN* Mutation (rs397515373, *c.373 + 5 G>A*)

This mutation is very rare, with an average prevalence of 0.0004% in the population. It was necessary to obtain 500,000 samples to detect a mutation once. In 2004, a paper describing a case of *MSTN* mutation in a child was published. In both the allelic copies of *MSTN*, the newborn boy had mutations that suppressed the synthesis of the functioning *MSTN* protein. The child was observed to have enlarged muscles of the thighs and upper extremities at birth. Ultrasonography of this child showed that the cross-section of the quadriceps femoris muscle was 7.2 SD, which was higher than the average (± standard deviation) value for 10 persons matched for age and gender. Moreover, the thickness of his subcutaneous fat was 2.88 SD below the average value of that of his peers. All reflexes of the child were normal, except for those associated with tendons. Interestingly, this mutation was also present in other members of this family. One of the relatives was extraordinarily strong, and the 24-year-old mother of the child was a professional athlete and had developed muscles, although to a lesser extent than her son. This study showed for the first time that the *MSTN* rs397515373 mutation (*c.373 + 5 G>A*) leads to an increase in muscle mass and strength [49].

#### 1.2.2. *MSTN A55T* Mutation (rs180565, 163 *G>A*)

*A55T* is important for the stability of the inhibitory activity of *MSTN* and affects the mature *MSTN* [50].

A study devoted to physical exercise reported that after 8 weeks of exercise with weights the subjects with *A55T* polymorphism *AT* and *TT* genotypes had greater muscle hypertrophy than those with *AA* genotype [51]. Studies have shown that *MSTN* polymorphisms can affect the skeletal muscle phenotype after exercise with weights. However, previous studies of *MSTN* SNPs associated with muscle hypertrophy in response to prolonged power-oriented strength exercises have not confirmed pronounced muscle hypertrophy after strength physical load [52].

Studies on an Asian sample set (n = 500) showed that the *A55T* polymorphism can affect the activity of *MSTN*, mass of skeletal muscles, and the amount of fat in the body. The results have shown that the *A55T* polymorphism determines the genetic predisposition to the development of excessive obesity and low muscle mass in Asians [53].

Chinese scientists found that people with the *MSTN A55T AT* + *TT* genotype showed a significant increase in the thickness of biceps (0.292 ± 0.210 cm, *P* = 0.03) but not of quadriceps (0.254 ± 0.198 cm, *P* = 0.07) compared to those of *AA* genotype carriers. Thus, the obtained results suggest a possible association between *A55T* polymorphism and muscle hypertrophy caused by strength training in Chinese individuals [51].

Korean researchers have found that the *A55T* polymorphism is associated with skeletal muscle recovery after strength training. The study sample included 48 young, healthy college students (age 24.8 ± 2.2 years, height 176.7 ± 5.3 cm, weight 73.7 ± 8.3 kg) who performed 50 repetitions in strength exercises. After strength exercises subjects with heterozygous *AT* showed significantly faster muscle recovery than those in the *AA* group (*P* = 0.042). These results prove that the *A55T* polymorphism *AT* genotype is associated with a faster recovery of skeletal muscle strength after intense strength exercise [54].

Turkish scientists failed to identify the relationship between the *A55T* polymorphism and the morphological data of arm wrestlers [51,54,55]. Moreover, no statistically significant relationships have been found in highly qualified athletes in endurance sports [56,57].

#### 1.2.3. Mutation of *MSTN E164K* rs35781413 (*c.490G>A, p.Glu164Lus*)

In a number of studies related to the influence of this genotype on the phenotype of athletes and people not engaged in sports, the results of experiments showed no statistically significant differences [58]. According to the website http://www.ensembl.org (accessed on 15 October 2022), the average frequency of a rare allele was 1%.

There are only indirect assumptions that this mutation can affect the manifestation of muscle mass and strength in humans. These assumptions are based on the fact that this polymorphism can make a significant contribution to the biochemical variability of mature *MSTN*, and accordingly, affect the state of the vertebrate muscular system. However, this assumption requires further study [9,47].

#### 1.2.4. Mutation of *MSTN K153R* (rs1805086, *p.Lys153Arg, c.458A>G*)

The *MSTN* rs1805086 *RR* genotype gene is more common in top-class weightlifting athletes [59]. Some researchers found a positive association between the *K153R* rs1805086 polymorphisms and the manifestation of strength abilities and muscle hypertrophy [13,46,51,60], whereas other researchers did not find any significant connection [33,46,61]. Some studies have proven a connection with high performance in high jumps (*P* < 0.05) [46]. Studies on the relationship between *K153R* and skeletal muscle phenotypes in elderly Caucasian women have shown that the heterozygote *MSTN* rs1805086 *KR* is a favorable polymorphism for the increased muscle mass in the biceps of the shoulder [62].

In the studies conducted with 16 women and 34 men of Caucasian, African–American, and Afro–European ethnicities that participated in the European Championships and the Olympic games in sports, such as football (n = 4), basketball (n = 10), tennis (n = 6), volleyball (n = 6), canoeing (n = 2), rugby (n = 10), baseball (n = 6), and track-and-fields (sprint, javelin, and shot put) (n = 6), who were compared with a control group of 100 people, including 40 women and 60 men not involved in sports, the authors failed to find statistically significant differences between the elite athletes and people in the control group in terms of the *K153R* polymorphism occurrence frequency and the success in competitions [60].

Studies on the relationship between *MSTN* and muscle pathologies in healthy elderly people are contradictory [63]. The association between low *MSTN* levels and low skeletal muscle mass was observed only in men but not in women. The authors point to the need for further research on *MSTN* as a biomarker of muscle mass and strength [20].

#### 1.2.5. *MSTN K153R* (rs1805086) Polymorphism Frequency

**Figure 3:**
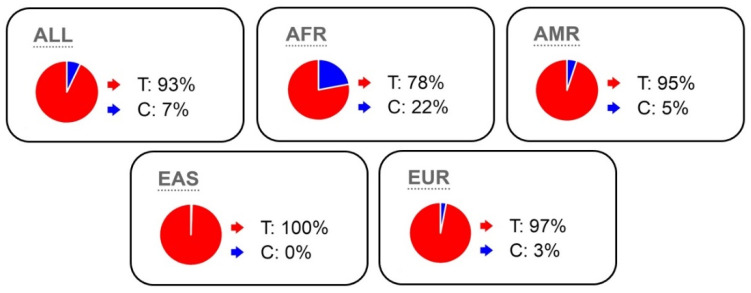
*T* and *C* allele frequencies of the *MSTN K153R* polymorphism according to the Ensembl database (All-General picture; AFR- African; AMR- American; EAS- Asian; EUR- Caucasian).

According to the Ensembl database, the frequency of the rare variant *K153R* is on an average 7% (3% in Caucasians and 22% in Africans), and large sample sizes are necessary to reliably identify the association of this polymorphism with strength abilities and muscle hypertrophy (Figure 3).

The conducted studies could not always prove a connection between the athletes’ skeletal muscle strength, muscle mass, and competitive performance [32,61]. Due to the low frequency of *K153R* polymorphism in Caucasian athletes of cyclic sports, the authors point out the possibility of evaluating *MSTN K153R* polymorphism during sports selection and that this mutation needs further study.

The problem with studying mutations in *MSTN* is the low frequency of some alleles. Obtaining the required number of subjects and statistically significant data would need very specific subjects, for example highly qualified athletes of weightlifting sports or people with an exceptional proportion of skeletal muscles [56]. Such subjects, for instance, can also include some ethnicities, considering their residence [33].

## 2. Materials and Methods

### 2.1. META-ANALYSIS

#### 2.1.1. Goal of Research

The purpose of this study was to summarize the relationship between *K153R* polymorphism and athletes’ strength indicators by conducting a systematic review and meta-analysis, which can potentially reveal more statistically reliable data compared to individual studies.

#### 2.1.2. Search for Publications

The search for scientific publications was carried out using PubMed, Web of Science, eLIBRARY.ru, SNPedia, Wiley Online Library, and Europe PMC resource databases. For this, the following keywords were used: myostatin, *MSTN*, *GDF-8*, *K153R*, and rs1805086. A list of publications on *MSTN* was compiled to include 71 scientific articles published prior to April 2021 from the above databases. For the analysis and systematization of publications, the EndNote Viever X9.2 bibliographic managers of Clarivate Analytics and the Zotero application were used. In addition to analyzing the content of the publications, their references were also studied. After selecting all potentially relevant articles, all the information on the *MSTN K153R* rs1805086 polymorphism was carefully studied in the control and experimental groups.

#### 2.1.3. Inclusion and Exclusion Criteria

Out of 71 scientific articles in PubMed, Web of Science, eLibrary.ru, SNPedia, Ensembl, Wiley Online Library, and Europe PMC databases, the publications directly related to the study of the *K153R* (rs1805086) polymorphism were selected. The studies used were marked with the following statement: “The study was approved by the ethics committee.”

To be included in this review, the studies had to meet the following criteria:

1. Be published from 1997 to April 2021,
2. Sample size should not be less than 10 people, and a control group in the study is mandatory,
3. Full text of the study should be available,
4. Participants must be adults who are not elderly,
5. Subjects had to be healthy at the time of the study,
6. No studies should be conducted on animals.

Of the 71 scientific articles, 61 were excluded after the first stage of working with databases. The criteria for rejecting the work included discrepancy between the title and subject of the study, experiments on animals, and experiments with small samples. The main criterion for dropping out of publications in the first stage was that the studies were not related to the *MSTN K153R* polymorphism.

**Figure 4:**
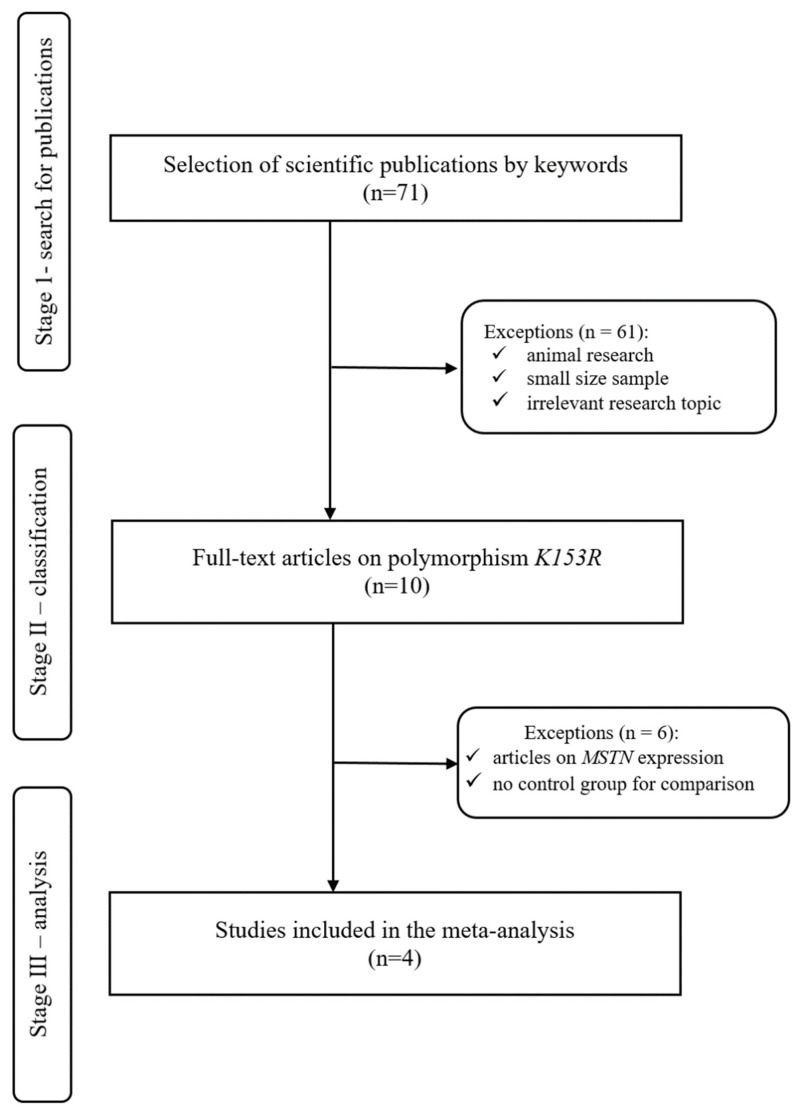
A flowchart representing the algorithm for selecting publications included in the meta-analysis.

After analyzing ten full-text publications, six were excluded after the second stage. These articles were devoted either to the expression of *MSTN* or they had an inadequate methodological quality of experiments. As a result, four publications were included in the meta-analysis. A flowchart showing the algorithm for selecting publications for the meta-analysis is shown in Figure 4.

### 2.2. Data Extraction

To include the publication in the meta-analysis, the full text of each study was analyzed for the general content and its compliance with the aforementioned acceptance criteria. From each eligible article, the following data were obtained: authors, publication year, study organization, study population (number of subjects, ethnicity, gender), polymorphism number and name, and muscle phenotype.

The phenotypic data included in this analysis were skeletal muscle mass and muscle strength.

### 2.3. Statistical Analysis

The Review Manager 5.4.1 (RevMan) computer program was used to perform the meta-analysis proposed by the Cochrane Community in 2014. The meta-analysis used data on the number of genotypes in the control and experimental groups to check for “publication error” (a systematic error associated with the predominant publication of positive results), and the asymmetry of the funnel graph [64]. The relationship between the *K153R* polymorphism and phenotypic data and the strength abilities of the subjects were evaluated using the odds ratio (OR) criterion and 95% confidence interval (CI) by comparing the control and experimental groups. The heterogeneity of the obtained data was estimated with the heterogeneity index I2 [65]. In the test for the overall effect, which was set according to the Z-criterion, the two-sided value of *P* was considered significant.

The statistical significance of each study included in the meta-analysis was also analyzed to compare the significance of this study with the generalized indicators. The degree of statistical significance of each study was assessed by χ^2^, in which *P* values < 0.05, were considered statistically significant. Statistical analysis according to the χ^2^-criterion was carried out using SPSS 23.0.

Based on the fact that in some publications, the results of the genotyping of subjects were expressed in nucleotides, whereas in others as amino acids, the following designations were used: the amino acid “*Lys*” was designated by the letter “*K*”, and “*Arg*” by the letter “*R*”. Thus, the mutant variant was designated by the letter “*R*”.

Problems with Sample Collection

Because the occurrence of the *MSTN K153R* rs1805086 polymorphisms in populations is 7% on average, it creates problems in identifying people with the rare genotype [32]. Two of the factors that significantly affect the association of *MSTN* genotypes with muscle mass and skeletal muscle strength are sex and age. Experiments to identify the effect of *MSTN* on muscle mass and strength showed different results depending on these two factors [66,67].

Contradictory data were also obtained in cases where the subjects were representative of different sports [56]. The impact of *MSTN* on skeletal muscle strength depends on the sport. In the type of sport requiring the ability to maintain a given physical load for a long time, no statistically significant data were found on the association of *MSTN* polymorphisms with muscle mass and strength [61].

Some studies have reported that estrogen affects the change in the expression of *MSTN* caused by power-oriented physical exercises [66]. In addition, differences in ethnicity, sample size, body weight, and level of physical activity may be potential reasons for the different results in studies related to *MSTN* [68]. The authors pointed out that nutritional factors should be considered when assessing the level of *MSTN*. Sex is also an important factor in the reduction of muscle strength and age-related decrease in muscle mass. Men usually begin to lose muscle mass after 40 years of age, when the level of testosterone in serum drops. Women can gradually lose 10–15% of their muscle mass from age 25 until the onset of menopause, after which it increases at a rate of 2% annually. Therefore, the amount of muscle mass is also affected by sex and diet.

In addition, studies often consider a specific human muscle as an object, such as the biceps or quadriceps, and this is also a limiting factor for the full assessment of the relationship between *MSTN* and the muscle mass and strength of the entire body as a whole.

The absence of a control group in some studies does not allow the solution to the problem of the statistical validity of the obtained data [55]. To obtain the most reliable data, larger sample sizes are required.

Finally, after testing for strength exercises, researchers considered only some indicators of muscle fatigue; therefore, they were limited to confirming the connection mechanism between specific strength indicators, *MSTN* genotypes, and muscle strength. It should be noted that most scientific publications are primarily based on previous research [68,69,70].

There are studies in which the authors concluded that the *MSTN K153R* polymorphism does not affect muscle phenotypes in women, wherein their sample of subjects was 33 people aged between 90–97-years-old. Considering that this polymorphism is very rare, the results of such studies seem very doubtful [20].

Thus, in this manuscript, the systematic review of publications related to the influence of the *MSTN K153R* polymorphism helped conclude that the data obtained can be regarded as contradictory. With such a discrepancy, the question arises whether the allelic variants *K* and *R* of the *MSTN* rs1805086 gene are genetic factors that can affect human strength abilities and skeletal muscle hypertrophy.

Meta-analysis overcomes the limitation of a small sample size by combining the results of a number of individual studies to obtain a single best estimate.

## 3. Results

In this study, it was found that athletes with the *R MSTN* variant had significantly greater muscle strength and mass due to the power-oriented physical training compared with that of the carriers of the *K MSTN* variant. This indicates that the presence of the *R* variant in *MSTN* rs1805086 can be considered a genetic marker associated with increased skeletal muscle strength and muscle mass (OR = 2.02, *P* = 0.05).

### Case-Control Study

In the control group of African American athletes, the frequency of the *RR* rs1805086 genotype was higher in 2–3% of cases than in the control groups in Russian and Caucasus natives. The *KR* heterozygote was also more common in the African–American control group than in the Russian control group (Table 1). In addition, the rs1805086 *KR/RR* genotypes were also significantly more common in the African–American athletes (35% and 14% vs. 13.0% and 7.1%, respectively) (Table 2).

Checking the statistical significance of the obtained data by conducting a Chi-square test (χ^2^) for the analysis of each sample separately did not show any statistically significant results, with the exception of the Russian subjects of 2017 [71]; however, the generalization of the data yielded statistically significant results (*P* = 0.030) for the analysis of genotypes *KK*, *KR*, and *RR*, and *P* = 0.0011 for the genotypes *KR* and *KK/RR*.

The frequency of rs1805086 *KR/RR* genotypes was significantly higher in the group of athletes than that of those in the control group (Table 2). African–American athletes were an exception. This may be due to the fact that, according to the Ensembl resource, the frequency of *K153R* polymorphism worldwide is significantly higher among African–Americans (average of 22%) than in other populations (Figure 3).

**Figure 5:**
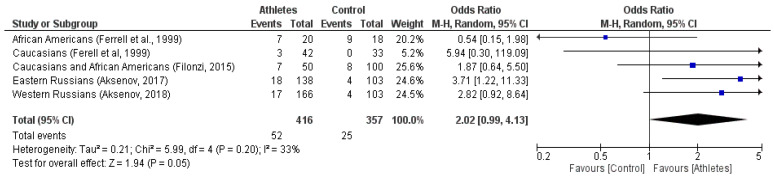
Meta-analysis of studies on the *MSTN K153R RR* polymorphisms association with skeletal muscle strength and muscle mass (random effect) [13,60,71,72].

**Figure 6:**
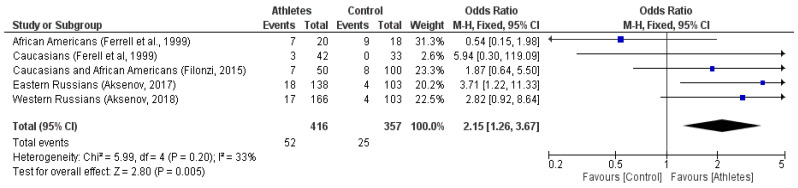
Meta-analysis of studies on the *K153R MSTN* polymorphisms association with skeletal muscle strength and muscle mass (fixed effect) [13,60,71,72].

In general, five ethnic sub-groups were used for the meta-analysis, in which 416 athletes and 357 subjects from the control group participated. The frequencies of the *KR/RR* genotypes compared to that of the *KK* genotype were significantly higher in the group of athletes (12.5%) compared to the control group (95% CI, *P* = 0.011). The results of the meta-analysis were as follows: the random effects model: OR = 2.02, *P* = 0.05, Z = 1.94 (Figure 5) and the fixed-effects model: OR = 2.15, 95% CI, *P* = 0.05; (Figure 6). The coefficient of heterochrony between the studies was I2 = 33% (*P* = 0.20). These results show that the mutation of the *R* variant (i.e., the *KR/RR* genotypes) has a statistically significant relationship with the phenotype of athletes with respect to the development of skeletal muscle strength abilities and muscle mass. The odds ratio in the meta-analysis was assessed by the Mantel-Haenszel (M-H) test. The funnel plot for the M-H criterion is shown in Figure 5 and Figure 6.

The hypothesis that the effectiveness of strength training is significantly higher in athletes with the *R MSTN* rs1805086 variant was confirmed by a meta-analysis (Figure 5 and Figure 6).

## 4. Discussion

As mentioned earlier, the frequency of mutant homozygotes (*RR*) is below 1% among the general population, which limits the possibility of studying large groups of people with variant *R*. On the other hand, according to Ensembl base, the frequency of the mutant variant *R* is approximately 3–4% on average among the general population across the globe. Among the athletes of power and weightlifting sports, the frequency of the minor variant *R* and the homozygote *RR* was significantly higher, reaching 10% [73].

The *K153R* polymorphism is significant in the development of muscle mass and strength. Previously, a number of studies have also shown that the rare *R* variant increases the inhibition of *MSTN* synthesis, thereby leading to an increase in skeletal muscle mass and muscle strength [37].

When searching for publications in several databases, four suitable studies were found in which athletes were compared with subjects in a control group. The feature of comparison was the *MSTN K153R* polymorphism. After combining the data from the selected studies, the group of athletes with the rare *R* variant comprised 52 subjects, and the control group comprised 25 subjects (12.5% vs. 7.9%, respectively). The generalized data showed statistically significant differences (*P* = 0.011) according to the χ^2^-criterion. The rare occurrence of this allele did not allow the obtaining statistically significant differences separately, with the exception of a sample of Eastern Russians [71,72]. Combining the samples into a single general population made it possible to increase the level of statistical significance of the analyzed data.

The data reported were not included in the meta-analysis, in which the author studied 79 Turkish athletes (arm wrestlers) aged 24 years in comparison with a control group consisting of 34 people. Associative studies were conducted on two polymorphisms of *MSTN*, *A55T*, and *K153R*. The authors failed to find statistically significant relationships between the studied polymorphisms and anthropometric indicators. Perhaps the reason for such data was the absence of genotypes with the *R* variant among the group of athletes [55].

In another study, the authors found that the currently published data on the *MSTN K153R* polymorphism and the human muscle phenotypes show contradictory results [66]. A number of studies have reported a significant effect of *MSTN* variants, and the reaction of muscle mass in response to strength training regardless of sex, which confirms the hypertrophic response to strength training in adults of both sexes. The *K153R* polymorphism is associated with a greater muscle hypertrophic response to exercise [74].

A study of the relationship between the *MSTN K153R* polymorphism and “explosive” leg strength in untrained men proved that the *MSTN K153R* polymorphism is associated with the ability to generate “peak” power during muscle contractions, evaluated using the vertical jump test [46]. The authors indicated that the polymorphisms *Lys (K)* and 153 *Arg (R)* located in exon 2 (replacement rs1805086, 2379 *A>G*) affect the phenotype of skeletal muscles. The replacement of the amino acid *Lys (K)* with *153 Arg (R)* was found in the active mature peptide of the *MSTN* protein, and this replacement can affect proteolytic processing due to its propeptide or the ability to bind to *ActRIIB*, which in turn, induces myoblast proliferation and muscle mass differentiation.

A study conducted in China on 94 healthy untrained men of the age group 8–22 years, convincingly demonstrated that the increase in the thickness of biceps ($\bar{X}$ = 0.300 ± 0.131 cm) and quadriceps ($\bar{X}$ = 0.421 ± 0.281 cm) (*P* < 0.01 for both muscle groups) was significantly higher among individuals with the *KR* genotype than among those with the *KK* genotypes of the *MSTN K153R* polymorphism. Thus, the obtained results proved that this polymorphism leads to larger skeletal muscle size in the absence of training and is also associated with a more noticeable increase in muscle mass after strength training in subjects with the *R* variant [51].

This is the first study to demonstrate the results of a meta-analysis of the *MSTN K153R* polymorphisms with the phenotype and functions of skeletal muscles in athletes. In particular, it was found that the frequency of genotypes that contribute to an increase in muscle volume and skeletal muscle strength in athletes (*KR* and *RR* genotypes) was significantly higher in the experimental group than in the control group. It was also confirmed that the *K153R* mutation is more common in the African–American group than that in other groups. In addition, a meta-analysis of five groups (two African–Americans, one Caucasus native, and two Russian), including a total of 773 test athletes and 357 general people in the control group, showed a significantly higher prevalence of *KR/RR* genotypes in the athletes than that in the control group.

The *R* variant is favorable for sports in which muscle strength and mass are important, such as bodybuilding, powerlifting, weightlifting, arm wrestling, kettlebell lifting, shot put, and bobsleigh. It can be assumed that the strong effect of this allele on the ability to become a successful athlete in weightlifting and speed-power sports is based on the inhibition of *MSTN* synthesis, as reported in some previous studies. In their respective studies, researchers have established a trend of *MSTN K153R* polymorphisms to influence the skeletal muscles’ hypertrophic response to strength training in women with the heterozygotic genotype [74]. The experiments have shown an increase in leg muscle mass in subjects with the *KR* genotype in response to strength training; this was 68% higher than in women with the *KK* genotype (*P* = 0.056) [74]. These data also indicate a significant role of rare variant *R* in *MSTN* in the hypertrophic response of the muscles of the subjects. The authors note that the *MSTN K153R* polymorphism has not been sufficiently studied and needs further research, particularly in women with high body weight. In addition, of interest is the reaction of the muscular system in response to power-oriented physical load, taking into account the *MSTN* genotypes.

It should also be noted that in almost all of the publications found, the authors indicated that the data obtained in the conducted studies may be limited by the levels of statistical significance of the statistical processing methods used. This is due to the low frequency of the *R MSTN* variant. Therefore, to solve this problem, further studies with larger sample sizes are needed. In addition, as in most studies related to the *K153R MSTN* polymorphism in athletes, it seems necessary to conduct experiments aimed at identifying associations between other *MSTN* polymorphisms and the expression of the *MSTN* protein in order to obtain additional information about the mechanisms by which *MSTN* polymorphisms affect the effectiveness of training process for increasing muscle mass and developing athletes’ strength abilities.

## 5. Conclusions

Meta-analysis of data on the *MSTN K153R* (rs1805086) polymorphisms convincingly demonstrated that the *KR* and *RR* genotypes are statistically associated with the strength abilities of athletes and their muscle mass when performing power-oriented training. Combining efforts in a search for subjects with the rare *R MSTN* variant will allow us to obtain more significant information about the magnitude of the effect of this polymorphism during strength training. Other polymorphisms of *MSTN* and its molecular mechanisms should also be studied, which will allow us to understand the factors that contribute to an increase in muscle strength and mass much better.

A deeper understanding of the mechanisms that control the maintenance of strength abilities of skeletal muscles will help to increase the effectiveness of sports selection, add to the list of molecular markers of sports inclinations, and develop more effective methods for the development of athletes’ strength abilities.

It is well-known that the inhibition of *MSTN* expression leads to an increase in muscle mass and improves muscle regeneration. Perhaps future studies will need to be performed to explore the relationship between *MSTN* and stem cells, which will allow new data to be obtained on the molecular mechanisms by which *MSTN* affects the manifestation of the abilities of weightlifting athletes. A better understanding of the molecular mechanisms of *MSTN* inhibition, including power-oriented physical load, is likely to be a promising area for improving the professional skills of weightlifting athletes.

## Figures and Tables

**Table 1:** *MSTN K153R* polymorphisms frequency distribution in the athlete and control groups.

Group	Athletes				Control				χ^2^ *P*
	Genotype			n	Genotype			n	
	*KK*	*KR*	*RR*		*KK*	*KR*	*RR*		
Caucasus natives [13]	39	3	0	42	33	0	0	33	-
African–Americans [13]	13	7	0	20	9	6	3	18	0.157
Caucasus natives, African–Americans, and Maori [60]	43	7	0	50	92	6	2	100	0.166
Eastern Russians [71]	120	4	14	138	99	4	0	103	0.004 *
Western Russians [72]	149	16	1	166	99	4	0	103	0.155
Generalized data	364	37	15	416	332	20	5	357	0.030 *

* *P*  < 0.05: statistically significant differences in the *R* variant frequency between the athlete and control groups.

**Table 2:** *MSTN K153R* polymorphism distribution in the athlete and control groups.

Group	Athletes				Control				χ^2^ *P*	link
	Genotype			n	Genotype			n		
	*KK*	*KR/RR*			*KK*	*KR/RR*				
Caucasus natives	39	3	(7.1%)	42	33	0	(-)	33	0.118	[13]
African–Americans	13	7	(35.0%)	20	9	9	(50.0%)	18	0.700	[13]
Caucasus natives, African–Americans, and Maori	43	7	(14.0%)	50	92	8	(8.0%)	100	0.249	[60]
Eastern Russians	120	18	(13.0%)	138	99	4	(3.9%)	103	0.015 *	[71]
Western Russians	149	17	(10.2%)	166	99	4	(3.9%)	103	0.059	[72]
Generalized data	364	52	(12.5%)	416	332	25	(7.9%)	357	0.011 *	

* *P*  < 0.05: statistically significant differences in the *R* variant frequency between the athletes and control groups.

## Data Availability

Not applicable.
